# Arrachements des épines iliaques antéro-supérieures et antéro-inférieures chez l'adolescent sportifs: à propos de deux cas

**DOI:** 10.11604/pamj.2015.22.356.8192

**Published:** 2015-12-11

**Authors:** Jamal Louaste, Taoufik Cherrad, Khalid Rachid

**Affiliations:** 1 Service de Chirurgie Orthopédique et Traumatologique, Hôpital Militaire Moulay Ismail, BP 50000 Meknès, Maroc

**Keywords:** Fracture-avulsion, épines iliaque antéro-supérieur, épines iliaque antéro-inférieur, adolescent, sport, Avulsion fracture, anterosuperior iliac spines, iliac spines anteroinferior, adolescent, sport

## Abstract

Les arrachements des épines iliaques antéro-supérieures et antéro-inférieures sont des entités rares qui touchent surtout l'enfant et l'adolescent. Elles se voient généralement lors d'une activité sportive. Le tableau clinique est dominé par une douleur brutale et importante de la hanche de type mécanique. Alors que le diagnostic est confirmé par les examens radiologiques. Nous rapportons deux cas de fractures arrachement des épines iliaques l'une antéro-supérieure et l'autre antéro-inférieure.

## Introduction

Les fractures avulsions des apophyses du bassin chez l'enfant et l'adolescent sont des affections peu communes, qui surviennent à l'effort physique. Elles sont responsables des douleurs aigües de la hanche et souvent confondues à des lésions tendineuses et des déchirures musculaires. Le diagnostic est orienté par la clinique et confirmé par la radiologie. Quant au traitement, il est essentiellement orthopédique. Nous rapportons deux cas de fractures arrachement des épines iliaques l'une antéro-supérieure et l'autre antéro-inférieure.

## Patient et observation

**Figure 1:**
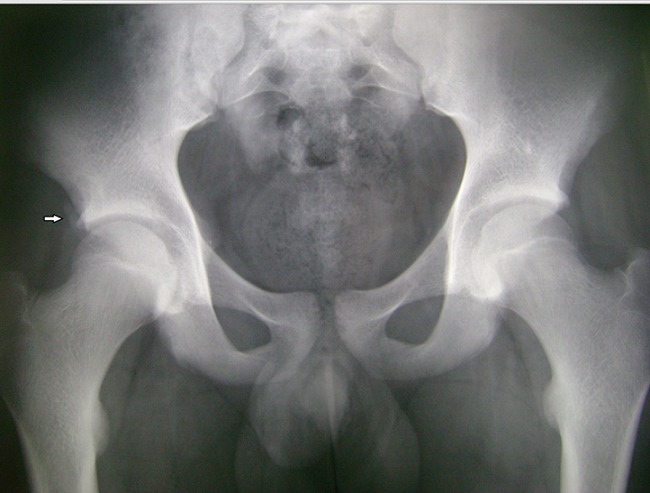
Radiographie du bassin face montrant un arrachement de l'EIAI

**Figure 2:**
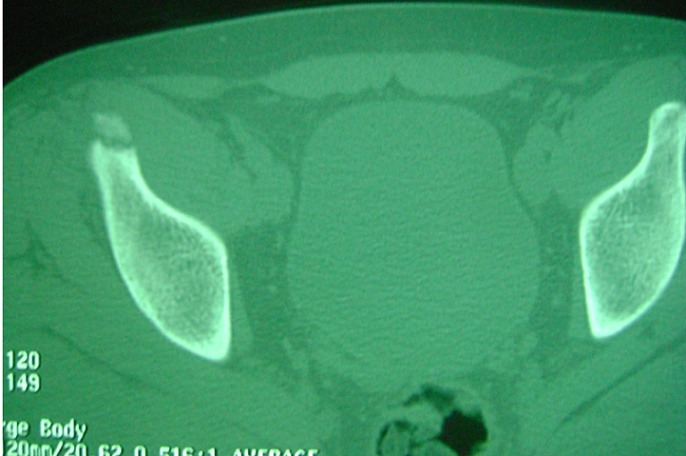
Tomodensitométrie en coupe axiale montrant une fracture arrachement de l'EIAI

**Figure 3:**
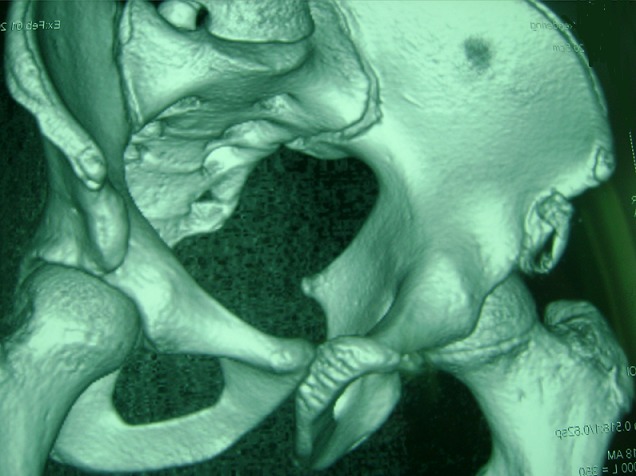
Tomodensitométrie avec reconstruction 3D montrant une fracture arrachement de l'EIAI

**Observation 1**: notre premier patient est un garçon âgé de 16 ans, sans antécédents pathologiques notables, qui a présenté lors d'un sprint une douleur vive de la hanche droite, associée à une impotence fonctionnelle immédiate. L'examen clinique a retrouvé, à l'inspection, l'absence de déformation évidente, d'ecchymose ou d'hématome. A la palpation, il y avait une sensibilité de la région inguinale droite. La hanche droite était non limitée mais sensible à la mobilisation. La flexion contre résistance de la hanche droite était également sensible. La radiographie standard du bassin face (Figure 1) a retrouvé une fracture-arrachement de l’épine iliaque antéro-inférieure (EIAI). Une tomodensitométrie du bassin a confirmé les données de la radiographie en montrant un arrachement de l'EIAI droite (Figure 2 et Figure 3). Le patient a été traité orthopédiquement par un repos en décubitus dorsal avec hanche droite fléchie par une attelle de boppe pendant dix jours. La mise en charge a été progressive pendant trois semaines aidée par des cannes anglaises. La reprise sportive a été autorisée à partir du troisième mois. L’évolution a été marquée par la diminution rapide de la douleur à deux semaines et la disparition totale des symptômes à un mois. Par ailleurs, aucunes complications n'ont été notées.

**Figure 4:**
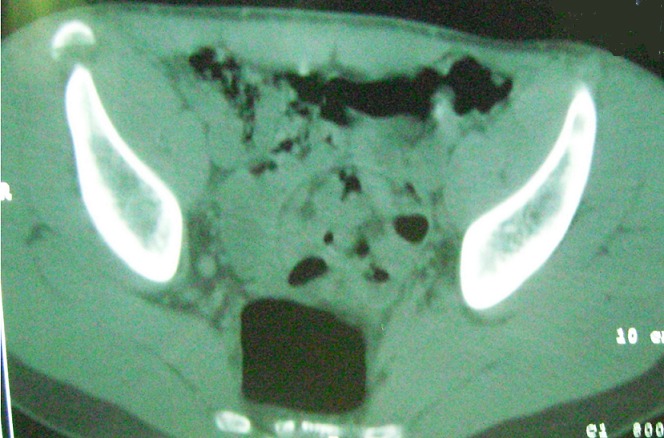
Tomodensitométrie en coupe axiale montrant une fracture arrachement de l'EIAS

**Figure 5:**
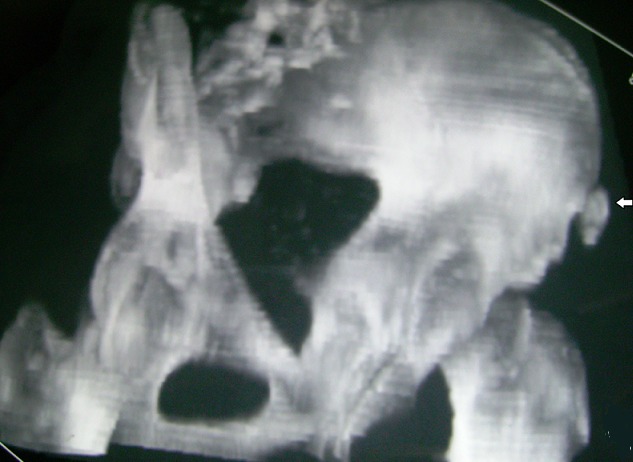
Tomodensitométrie avec reconstruction 3D montrant une fracture arrachement de l'EIAS

**Observation 2**: notre deuxième patient est un jeune âgé de 19 ans, sans antécédents pathologiques notables, qui a présenté lors d'un démarrage une douleur vive de la hanche gauche, associée à une impotence fonctionnelle immédiate. L'examen clinique a retrouvé, à l'inspection, l'absence d'ecchymose ou d'hématome. A la palpation, il y avait une sensibilité de l’épine iliaque antéro-supérieure (EIAS) gauche et de la région située en dessous. La mobilisation de la hanche est sensible. La flexion abduction contre résistance de la hanche gauche est douloureuse. Par ailleurs on ne note pas de trouble de la sensibilité de la cuisse. La radiographie standard du bassin n'a pas été concluante. Alors une tomodensitométrie du bassin a été demandée et elle a objectivé un arrachement de l'EIAS gauche (Figure 4 et Figure 5). Le traitement a été orthopédique et identique à celui du premier patient. Les résultats ont été bons avec disparition de la douleur après quelques semaines et une reprise des activités sportives après trois mois. Il n'y a pas eu de complications.

## Discussion

L'adolescence constitue la période d'ossification des noyaux apophysaires pelviens, au cours de laquelle les chaînes musculo-squelletiques sont soumises à des contraintes importantes notamment lors de l'activité sportive. Lors d'une contraction brutale (démarrage, shoot, sprint), ces noyaux représentent les maillons faibles de ces chaînes. Ils se rompent et donnent les fractures avulsions [1]. L'arrachement de la tubérosité ischiatique est la plus fréquente au niveau du bassin, suivi de celle de l'EIAS puis de l'EIAI [2]. Les lésions des épines iliaques sont rares et généralement unilatérales [3]. Ces arrachements surviennent chez l'adolescent et l'adulte jeune surtout de sexe masculin au cours d'activités sportives [4]. L'arrachement de l'EIAI est secondaire à une contraction brutale du muscle droit fémoral. Ceci est observé lors d'une extension de la hanche avec genou fléchi (shoot), ou lors d'une hyperextension de la hanche pendant un saut ou la poussée postérieure lors d'un sprint par exemple [3]. L'arrachement de l'EIAS succède à une contraction ou à un étirement brutal du muscle sartorius ou du tenseur du fascia lata, lors d'une extension brutale ou flexion contrariée de la cuisse, lors de l'armé du shoot par exemple ou le démarrage d'un sprint. Il faut toujours vérifier la sensibilité de la face antéroexterne de la cuisse, afin d’éliminer une exceptionnelle atteinte du nerf cutané latéral de la cuisse associée [5]. Cliniquement, ces arrachements se manifestent par des douleurs brutales et importantes de la hanche de type mécaniques. Une impotence fonctionnelle et une boiterie sont notées ainsi qu'un craquement audible peut être recherché dans l'anamnèse. L'examen physique retrouve une douleur à la mobilisation passive de la hanche et à la palpation de l’épine arrachée. Un signe très évocateur est représenté par le déclenchement de la douleur à l’étirement passif du muscle concerné et à sa contraction contrariée lors des tests isométriques. Vue leurs incidences, ces lésions sont souvent méconnues et confondues avec des lésions musculotendineuse d'où l'intérêt de confirmation par les examens paraclinique [3]. La radiographie standard du bassin face et les incidences ¾ permettent de poser le diagnostic, de déterminer la taille de l'apophyse arrachée et de mesurer le déplacement. Ce déplacement est généralement entre 2mm et 8mm [6]. La TDM avec des reconstructions 2D et 3D donne plus de précisions sur la taille et le déplacement du fragment. L’échographie constitue également un moyen de diagnostic, elle permet le dépistage précoce même en absence de centre d'ossification [7]. Le traitement orthopédique constitue la référence. Il repose sur le repos au lit hanche fléchie pendant 10 jours associé à un traitement antalgique. La remise en charge se fait progressivement en trois semaines, aidées par des cannes anglaises, puis l'appui est libre. L'activité sportive est reprise vers le troisième mois environ. Ce traitement donne des résultats fonctionnels très satisfaisant dans la majorité des cas. Le massage et la rééducation active sont à proscrire avant la huitième semaine car ils sont pourvoyeurs d'ossifications ectopiques [1]. La chirurgie est réservée aux rares formes très déplacées. Généralement, on opère les formes déplacées de plus de 2cm selon Lefort [6]. Les exostoses et les pseudarthroses représentent les principales complications du traitement orthopédique [8]. Alors que la méralgie parésthesique, le sepsis et la fracture du matériel d'ostéosynthése constituent les complications du traitement chirurgical [9].

## Conclusion

Les fractures-avulsion des apophyses du bassin sont des affections rares et souvent méconnues. Leurs diagnostic doit être évoquer devant l’âge jeune, les circonstances de survenue, ainsi que la symptomatologie clinique. La radiologie permet de confirmer ces fractures arrachements, dont le traitement est orthopédique essentiellement. La prévention de ces fractures-avulsions repose sur les échauffements et les étirements musculaire avant les efforts physiques de mêmes qu'un diagnostic précoce lors de leurs survenue ce qui évite une escalade inutile des examens complémentaires, et permet un traitement rapide et adéquat.
